# Preoperative prediction of microsatellite instability status in colorectal cancer based on a multiphasic enhanced CT radiomics nomogram model

**DOI:** 10.1186/s12880-024-01252-1

**Published:** 2024-04-02

**Authors:** Xuelian Bian, Qi Sun, Mi Wang, Hanyun Dong, Xiaoxiao Dai, Liyuan Zhang, Guohua Fan, Guangqiang Chen

**Affiliations:** 1 Department of Radiology, The Second Affiliated Hospital of Soochow University, San Xiang Road No. 1055, 215004 Suzhou, Jiangsu China; 2 Department of Pathlogy, The Second Affiliated Hospital of Soochow University, San Xiang Road No. 1055, 215004 Suzhou, Jiangsu China; 3 Department of Radiotherapy, The Second Affiliated Hospital of Soochow University, San Xiang Road No. 1055, 215004 Suzhou, Jiangsu China

**Keywords:** Microsatellite instability, Colorectal cancer, Radiomics, Multiphasic enhanced CT

## Abstract

**Background:**

To investigate the value of a nomogram model based on the combination of clinical-CT features and multiphasic enhanced CT radiomics for the preoperative prediction of the microsatellite instability (MSI) status in colorectal cancer (CRC) patients.

**Methods:**

A total of 347 patients with a pathological diagnosis of colorectal adenocarcinoma, including 276 microsatellite stabilized (MSS) patients and 71 MSI patients (243 training and 104 testing), were included. Univariate and multivariate regression analyses were used to identify the clinical-CT features of CRC patients linked with MSI status to build a clinical model. Radiomics features were extracted from arterial phase (AP), venous phase (VP), and delayed phase (DP) CT images. Different radiomics models for the single phase and multiphase (three-phase combination) were developed to determine the optimal phase. A nomogram model that combines clinical-CT features and the optimal phasic radscore was also created.

**Results:**

Platelet (PLT), systemic immune inflammation index (SII), tumour location, enhancement pattern, and AP contrast ratio (ACR) were independent predictors of MSI status in CRC patients. Among the AP, VP, DP, and three-phase combination models, the three-phase combination model was selected as the best radiomics model. The best MSI prediction efficacy was demonstrated by the nomogram model built from the combination of clinical-CT features and the three-phase combination model, with AUCs of 0.894 and 0.839 in the training and testing datasets, respectively.

**Conclusion:**

The nomogram model based on the combination of clinical-CT features and three-phase combination radiomics features can be used as an auxiliary tool for the preoperative prediction of the MSI status in CRC patients.

**Supplementary Information:**

The online version contains supplementary material available at 10.1186/s12880-024-01252-1.

## Introduction

Colorectal cancer (CRC) is currently the third most prevalent malignancy and the second most deadly cancer worldwide [1]. Microsatellites (MSs) are DNA sequences of a few nucleotides (typically 1–6) in the genome that are repeated in tandem [2]. DNA mismatch repair systems exist in normal organisms, and the most common DNA mismatch repair genes are MLH1, MSH2, MSH6, and PMS2. When mutations occur in any of the DNA mismatch repair genes or when MLH1 promoter hypermethylation occurs, this leads to the accumulation of erroneous MS sequences, which is called microsatellite instability (MSI) [3, 4].

MSI is one of the molecules associated with the oncogenic pathway of CRC, and it has an incidence of 15% [5]. Although the incidence of MSI in CRC is low, it has special clinical significance. First, Lynch syndrome can be screened for primarily via MSI testing [6, 7]. Second, for early-stage CRC, especially in stage II, MSI status is a positive prognostic factor [8]. Finally, CRC patients with MSI status may benefit from immunotherapy but not from 5-FU-based chemotherapy regimens [9, 10]. Therefore, the detection of MSI status has certain clinical value in guiding the diagnosis, treatment and prognosis evaluation of CRC patients.

MSI detection is usually performed by invasive methods, such as polymerase chain reaction (PCR) and immunohistochemistry (IHC), to obtain pathological tissues. These methods are time-consuming and expensive, and biopsies are only able to obtain a very small fraction of lesions. Thus, it is difficult to adequately demonstrate the MS status of the tumour [11]. Therefore, a noninvasive, economical preoperative approach is required to predict MSI status in CRC patients.

In clinical work, enhanced CT is a frequently employed noninvasive examination method to determine the local and systemic conditions of CRC patients, which helps in disease diagnosis and treatment plan selection. However, traditional medical imaging mainly relies on the visual perspective to define features [12]. Thus, a large amount of image information is lost, and the identification of the MSI status of CRC patients remains challenging. In contrast, radiomics combines the quantitative analysis of medical images and machine learning methods. This approach can deeply mine a significant number of image data in medical images that cannot be identified by human visual perspective, providing more accurate information for medical imaging diagnosis and treatment [13–15]. However, radiomics are not foolproof and need to be complemented by combining clinical and medical image features [12]. Therefore, a comprehensive and efficient prediction model can only be constructed by combining multidimensional information such as radiomics, clinical and medical image features. At present, there are relatively few reports on the use of CT radiomics to predict MSI status in CRC patients [16–18]. Furthermore, these studies did not include sufficiently comprehensive clinical and CT features and only analysed radiomics features in the venous phase (VP). Therefore, the aim of this study was to build the clinical model, radiomics models, and nomogram model to preoperatively predict MSI status in CRC patients based on more comprehensive clinical-CT features and multiphasic enhanced CT radiomics.

## Materials and methods

### Patients

**Fig. 1:**
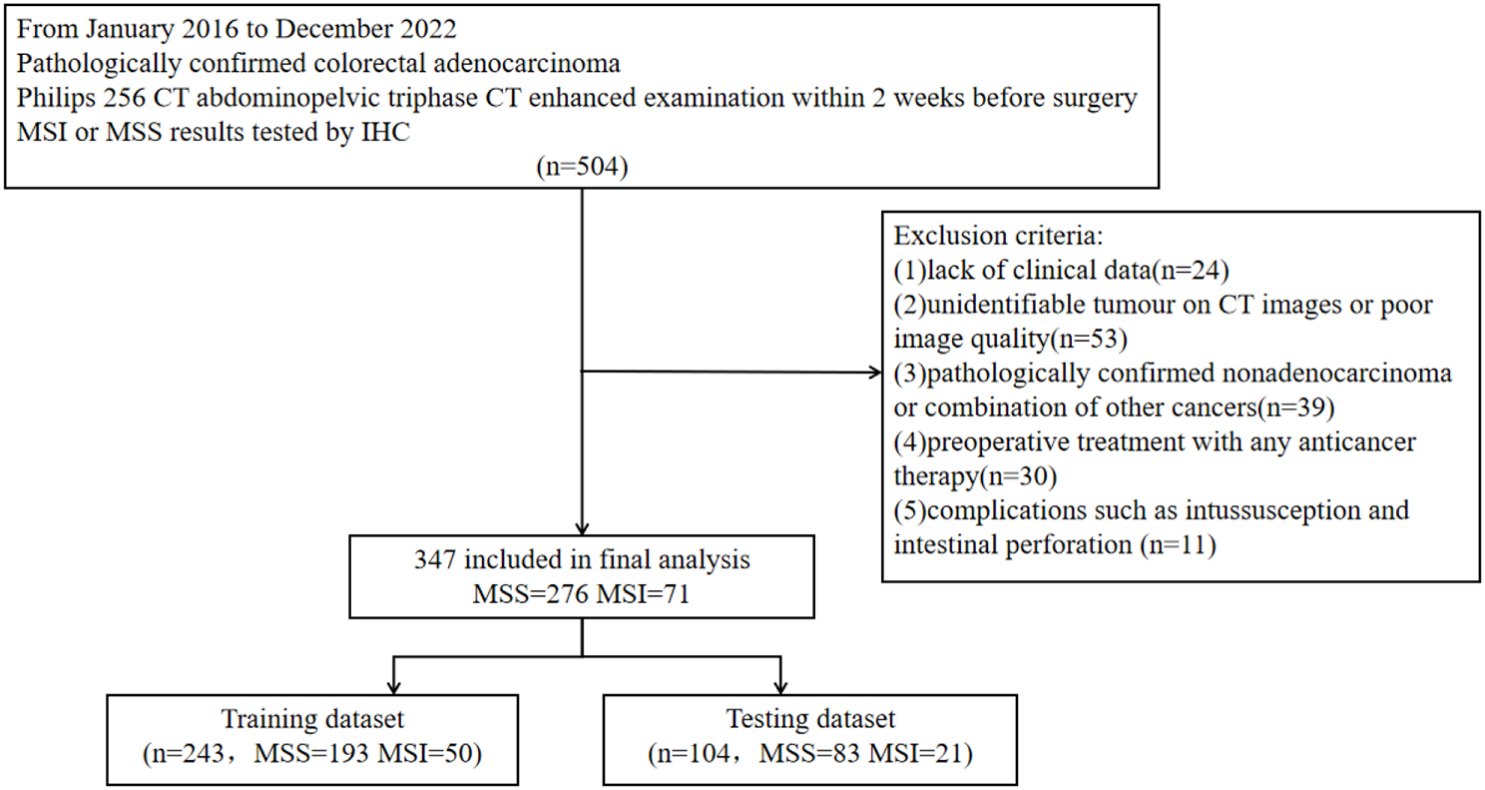
Patient screening flow chart

A total of 504 CRC patients who matched the inclusion criteria from January 2016 to December 2022 in our hospital were initially enrolled. Inclusion criteria: (1) pathologically confirmed colorectal adenocarcinoma; (2) Philips 256 CT abdominopelvic triphasic CT enhanced examination within 2 weeks before surgery; and (3) MSI or MSS results tested by IHC. Exclusion criteria: (1) lack of clinical data (*n* = 24); (2) unidentifiable tumour on CT images or poor image quality (*n* = 53); (3) pathologically confirmed nonadenocarcinoma or combination of other cancers (*n* = 39); (4) preoperative treatment with any anticancer therapy (radiotherapy, chemotherapy, biotherapy, etc.) (*n* = 30); and (5) complications such as intussusception and intestinal perforation (*n* = 11). Finally, 347 patients were enrolled, including 276 MSS patients and 71 MSI patients. These patients were randomly allocated to the training dataset, which had 243 participants, and the testing dataset, which had 104 participants. Figure 1 depicts the patient screening procedure.

### Microsatellite instability status assessment

MSI is caused by functional defects in DNA mismatch repair proteins (MLH1, MSH2, MSH6, and PMS2), and the deletion of DNA mismatch repair proteins was observed by IHC. Patients with negative staining for one or more of the four mismatch repair proteins were assigned to the MSI group, while those with positive staining for all four were assigned to the MSS group [19].

### Clinical features

Clinical information within 1 week before surgery was collected by reviewing electronic cases of CRC patients. This information included general information, such as age, sex, smoking status, alcohol habits, family history of cancer, hypertension, and diabetes. This information also included laboratory indices, such as carcinoembryonic antigen (CEA), carbohydrate antigen 19 − 9 (CA199), white blood cell (WBC), neutrophil (NE), lymphocyte (LYM), platelet (PLT), C-reactive protein (CRP), and albumin (ALB) levels as well as the calculated neutrophil to lymphocyte ratio (NLR = NE/LYM) and systemic immune inflammation index [SII = PLT×(NE/LYM)].

### CT image acquisition and analysis

Every patient was examined using a Philips 256 CT scanner. Patients fasted for more than 8 h before the examination, and a total of approximately 1500 ml of negative contrast agent was administered orally approximately 2 h before the scan. Specific parameters: tube voltage, 120 kV; automated tube current; slice thickness, 5 mm; and matrix, 512 × 512. After the nonenhanced abdominal CT scan, 80–100 ml of iodine contrast was injected, and enhanced scans of the arterial phase (AP), VP, and delayed phase (DP) were performed at 25–35 s, 65–80 s, and 210 s after contrast administration, respectively.

Features on the CT images were as follows: (1) clinical T (cT) stage and clinical N (cN) stage: according to the AJCC 8th edition [20]; (2) tumour location: the right colon, including the proximal 2/3 transverse colon, ascending colon and caecum, and the left colon or rectum, including the distal 1/3 transverse colon, descending colon, sigmoid colon and rectum [21]; (3) tumour length; (4) maximum tumour diameter; (5) enhancement pattern: homogeneous refers to the difference between the largest and smallest CT value of the lesion in the VP is less than or equal to 10 Hounsfield units (HU), and heterogeneous refers to the difference between the largest and smallest CT value of the lesion in the VP is greater than 10 HU [22]; and (6) CT contrast ratio (CR): ratio of the CT value of the lesion to the CT value of the abdominal aorta or its branches at the same level [23], including the plain phase contrast ratio (PCR), AP contrast ratio (ACR), VP contrast ratio (VCR) and DP contrast ratio (DCR). CT value measurement: Examine the plain scan and the triphasic enhanced CT images of AP, VP, and DP, and choose the phase with the clearest tumour boundary as the baseline for outlining the ROI, avoiding the edge of the tumour by about 2 mm [24], avoiding fat, gas, intestinal contents, calcification, and so on. The ROI of the abdominal aorta or its large branches at the same level as the lesion was outlined, avoiding the vessel wall and plaque. To guarantee that the size and location of the areas are consistent from one phase to the next, the ROIs are created via copying and pasting. CT images of all patients were analyzed by a gastrointestinal radiologist with 3 years of experience, in the presence and under the guidance of a chief gastrointestinal radiologist with 25 years of experience, both of whom were unaware of the pathologic information of the CRC patients. In case of disagreement, negotiation was conducted to reach a consensus [25, 26]. Quantitative indicators were all measured three times and averaged.

### Image segmentation [25, 26]

The CT images of AP, VP and DP were imported sequentially into ITK-SNAP software (v3.8.0, http://www.itksnap.org) in DICOM format for each patient. A gastrointestinal radiologist with 3 years of experience outlined ROIs layer by layer on the triphasic enhanced CT images, which should include haemorrhage and necrotic areas and avoid fat, air and intestinal contents. If identifying the lesion’s border is challenging, it can be done by varying the window width and window level, or by doing multilevel and multidimensional observation. Then, a chief gastrointestinal radiologist with 25 years of experience reviewed and modified these images and generated a volume of interest (VOI) for the tumour. In case of disagreement, negotiation was conducted to reach a consensus. Neither physician had any knowledge of the pathology information of each CRC patient.

### Feature extraction and selection

FeAture Explorer (v0.5.5, https://github.com/salan668/FAE), an open-source radiomics analysis platform written in Python 3.7.6 [27], was used to extract the radiomics of each patient from 10 image types and 3 different feature systems. The 10 image types are Original, Wavelet Transform, Square, Square Root, Logarithm, Laplacian of Gaussian, Gradient, Exponential, Local Binary Pattern (2D), and Local Binary Pattern (3D). The three types of feature systems are first-order features, shape features, and texture features, where texture features include the gray-level cooccurrence matrix (GLCM), gray-level run-length matrix (GLRLM), gray-level size zone matrix (GLSZM), neighbouring gray tone difference matrix (NGTDM) and gray-level dependence matrix (GLDM). Finally, 1772 radiomics features were extracted from each of the AP, VP and DP images.

The above-extracted features were then screened. First, the synthetic minority oversampling technique (SMOTE) was utilized to correct the effects of the uneven sample sizes of the MSI and MSS groups. Second, the Z-score was used to normalize the data features. Third, the Pearson correlation coefficient (PCC) method was used for dimensionality reduction, and redundant features with PCC > 0.99 were removed. Fourth, recursive feature elimination (RFE) was used to select features. The goal of RFE is to progressively reduce the set of classifier-based features, with the range of feature numbers set from 1 to 20 [27, 28]. Fifth, a logistic regression classifier and fivefold cross validation were used.

### Model building and analysis

Using univariate and multivariate analyses, clinical-CT features associated with MSI status in CRC patients were screened out, and a clinical model was built. To determine the enhanced phase with the best MSI prediction performance, AP, VP, DP, and three-phase combination models were also built, and linear combination weights were calculated to form the radscore. To create a more comprehensive prediction model, the screened clinical-CT features and the best phasic radscore were integrated to build a nomogram model. The prediction effectiveness of each model was evaluated by the area under curve (AUC) of the receiver operating characteristic (ROC) curve, and the difference in AUC values among the models was compared using the DeLong test. The calibration curve was used to evaluate the agreement between the predicted and actual probabilities of MSI status by the nomogram, and the Hosmer‒Lemeshow test was used to evaluate the goodness of fit of the nomogram. Decision curve analysis (DCA) was performed to determine the clinical utility of each model by comparing the net benefit at different threshold probabilities. The flow chart of radiomics is shown in Supplementary Material Figure S1.

### Statistical analysis

SPSS 26.0 and R 4.3.0 were used to conduct the statistical analysis. Independent sample *t* test, the Mann-Whitney *U* test, and the chi-squared test were used to compare continuous and categorical variables. In a multivariate binary logistic regression, variables with statistically significant univariate analysis were added to identify independent risk factors related to MSI status in CRC patients. Waterfall plots, nomogram, ROC curves, calibration curves and DCA were plotted and analysed by R software. *P* < 0.05 indicates a statistically significant difference.

## Results

### Clinical features

This study comprised 347 individuals with CRC. Their ages ranged from 28 to 90 years, and the mean was 66 years. There were 200 males and 147 females. The MSI group had higher PLT and SII levels than the MSS group (*P* < 0.05). Other clinical features between the MSI and MSS groups, including age, sex, smoking, alcohol, family history of cancer, hypertension, diabetes, CEA, CA199, WBC, NE, LYM, CRP, ALB, and NLR, were not substantially different (*P* > 0.05). The incidences of MSI in the training and testing datasets were 20.58% (50/243) and 20.19% (21/104), respectively, with no significant differences in clinical features between the two groups (*P* > 0.05) (Table 1).

**Table 1:** Analysis of the clinical features of 347 patients with colorectal cancer [median (Q1, Q3) or no. (%)]

	MSI(*n* = 71)	MSS(*n* = 276)	P	Training(*n* = 243)	Testing(*n* = 104)	P
Age(y)	65.00(57.00,73.00)	66.00(59.00,74.00)	0.566	66.00(60.00,73.00)	64.00(55.25,74.75)	0.250
Sex,n(%)			0.804			0.989
Male	40(56.34)	160(57.97)		140(57.61)	60(57.69)	
Female	31(43.66)	116(42.03)		103(42.39)	44(42.31)	
Smoking,n(%)			0.117			0.104
Yes	8(11.27)	53(19.20)		48(19.75)	13(12.50)	
No	63(88.73)	223(80.80)		195(80.25)	91(87.50)	
Alcohol, n(%)			0.516			0.099
Yes	7(9.86)	35(12.68)		34(13.99)	8(7.69)	
No	64(90.14)	241(87.32)		209(86.01)	96(92.31)	
Family history of cancer, n(%)			0.949			1.000
Yes	2(2.82)	5(1.81)		5(2.06)	2(1.92)	
No	69(97.18)	271(98.19)		243(97.94)	104(98.08)	
Hypertension, n(%)			0.392			0.103
Yes	38(53.52)	132(47.83)		117(48.15)	44(57.69)	
No	33(46.48)	144(52.17)		126(51.85)	60(42.31)	
Diabetes, n(%)			0.612			0.603
Yes	12(16.90)	40(14.49)		38(15.64)	14(13.46)	
No	59(83.10)	236(85.51)		205(84.36)	90(86.54)	
CEA(ng/ml)	3.73(2.15,11.08)	4.23(2.37,16.73)	0.155	3.89(2.25,15.58)	4.57(2.49,12.05)	0.560
CA199(U/ml)	12.59(7.61,25.47)	13.13(7.49,23.19)	0.635	12.80(7.61,23.50)	14.48(6.86,23.19)	0.852
WBC(×10^9^/L)	6.10(5.20,7.30)	6.10(4.90,7.20)	0.535	6.10(5.10,7.20)	5.85(4.83,7.40)	0.648
NE(×10^9^/L)	3.90(2.90,4.80)	3.70(2.80,4.60)	0.221	3.70(2.90,4.60)	3.55(2.80,4.80)	0.751
LYM(×10^9^/L)	1.50(1.10,2.00)	1.50(1.20,1.90)	0.766	1.50(1.20,1.90)	1.50(1.10,1.90)	0.956
PLT(×10^9^/L)	270.00(214.00,301.00)	228.50(188.25,276.75)	0.002	230.00(192.00,280.00)	251.00(190.25,299.75)	0.203
CRP(mg/L)	5.60(5.40,6.00)	5.50(5.00,5.90)	0.085	5.50(5.10,5.90)	5.60(5.10,5.98)	0.416
ALB(g/L)	41.00(38.10,43.90)	41.35(37.90,44.10)	0.836	41.20(37.90,44.10)	41.80(37.90,44.03)	0.935
NLR	2.56(1.72,3.54)	2.43(1.67,3.47)	0.366	2.46(1.69,3.46)	2.46(1.60,3.53)	0.850
SII(×10^9^/L)	630.95(431.95,957.38)	539.02(362.42,816.78)	0.024	570.67(378.00,813.93)	566.59(370.90,938.09)	0.485

### CT features

Compared with that in the MSS group, the CRC in the MSI group was more prevalent in the right colon, with more heterogeneous enhancement and lower PCR, ACR, VCR, and DCR (*P* < 0.05). Other CT features, including cT stage, cN stage, tumour length, and maximum tumour diameter, did not substantially differ between the MSI and MSS groups (*P* > 0.05). There were no significant differences in CT features between the training and testing datasets (*P* > 0.05) (Table 2).

**Table 2:** Analysis of the CT features of 347 patients with colorectal cancer [median (Q1, Q3) or no. (%)]

CT features	MSI(*n* = 71)	MSS(*n* = 276)	P	Training(*n* = 243)	Testing(*n* = 104)	P
cT-stage, n(%)			0.106			0.096
T1	5(7.04)	7(2.54)		7(2.88)	5(4.80)	
T2	4(5.63)	33(11.96)		32(13.17)	5(4.80)	
T3	58(81.70)	213(77.17)		187(76.95)	84(80.78)	
T4	4(5.63)	23(8.33)		17(7.00)	10(9.62)	
cN-stage, n(%)			0.279			0.793
N0	38(53.52)	127(46.01)		118(48.56)	47(45.19)	
N1	17(23.94)	60(21.74)		54(22.22)	23(22.12)	
N2	16(22.54)	89(32.25)		71(29.22)	34(32.69)	
Tumour location, n(%)			< 0.001			0.817
Right colon	37(52.11)	80(28.99)		81(33.33)	36(34.62)	
Left colon or Rectum	34(47.89)	196(71.01)		162(66.67)	68(65.38)	
Tumour length(cm)	46.66(35.27,65.88)	48.06(36.40,62.69)	0.535	48.10(36.78,62.65)	46.33(35.27,63.67)	0.932
Maximum tumour diameter(cm)	18.10(15.08,26.24)	17.69(13.61,24.85)	0.142	17.54(13.67,24.96)	18.38(14.47,25.73)	0.420
Enhancement, n(%)			< 0.001			0.118
Homogeneous	40(56.34)	238(86.23)		200(82.30)	78(75.00)	
Heterogeneous	31(43.66)	38(13.77)		43(17.70)	26(25.00)	
PCR	0.89(0.82,0.96)	0.92(0.84,1.02)	0.009	0.91(0.83,1.02)	0.92(0.84,1.00)	0.630
ACR	0.21(0.16,0.24)	0.23(0.21,0.26)	< 0.001	0.23(0.20,0.26)	0.22(0.20,0.25)	0.143
VCR	0.46(0.38,0.50)	0.51(0.47,0.56)	< 0.001	0.50(0.45,0.55)	0.51(0.45,0.56)	0.883
DCR	0.64(0.58,0.69)	0.68(0.63,0.73)	< 0.001	0.67(0.63,0.72)	0.68(0.60,0.73)	0.854

### Clinical model building and analysis

The indicators that were statistically significant in the univariate regression analysis, including PLT, SII, tumour location, enhancement pattern, PCR, ACR, VCR, and DCR, were included in the multivariate regression analysis, and this analysis revealed significantly different results for PLT, SII, tumour location, enhancement pattern, and ACR (*P* < 0.05) (Table 3). We constructed clinical models based on the above five clinical CT features, and the AUCs were 0.765 (95% CI: 0.687–0.843) and 0.783 (95% CI: 0.642–0.923) in the training and testing datasets, respectively.

**Table 3:** Multivariate regression analysis of clinical-CT features

Clinical-CT features	OR	95% CI	P
PLT,per 100 × 10^9^/L	1.458	1.011–2.103	0.043
SII,per 100 × 10^9^/L	1.051	1.011–2.103	0.034
Tumour location	2.039	1.100-3.778	0.024
Enhancement	2.176	1.079–4.391	0.030
PCR,per 0.1	0.791	0.621–1.006	0.056
ACR,per 0.1	0.394	0.168–0.925	0.032
VCR,per 0.1	0.594	0.332–1.061	0.079
DCR,per 0.1	0.859	0.528–1.395	0.539

### Radiomics model building and analysis

AP, VP, DP, and three-phase combination models were constructed by extracting 1772 radiomics features from each patient’s AP, VP, and DP images, respectively. Finally, 10 AP, 7 VP, 7 DP, and 12 three-phase combination radiomics features were retained, respectively. The radiomics features screened in each phase are shown in Supplementary Material Table S1.

Four radiomics models were constructed, and the AUCs of the AP model, VP model, DP model, and three-phase combination model in the training dataset were 0.772 (95% CI: 0.701–0.843), 0.722 (95% CI: 0.645–0.799), 0.750 (95% CI: 0.679–0.821) and 0.827 (95% CI: 0.763–0.891), respectively. The AUCs in the testing dataset were 0.760 (95% CI: 0.638–0.881), 0.671 (95% CI: 0.552–0.790), 0.668 (95% CI: 0.532–0.805), and 0.714 (95% CI: 0.604–0.825), respectively. In the training dataset, the three-phase combination model had the highest predictive efficacy. Therefore, the three-phase combination model was ultimately selected as the best radiomics model.

### Nomogram model building and analysis

**Fig. 2:**
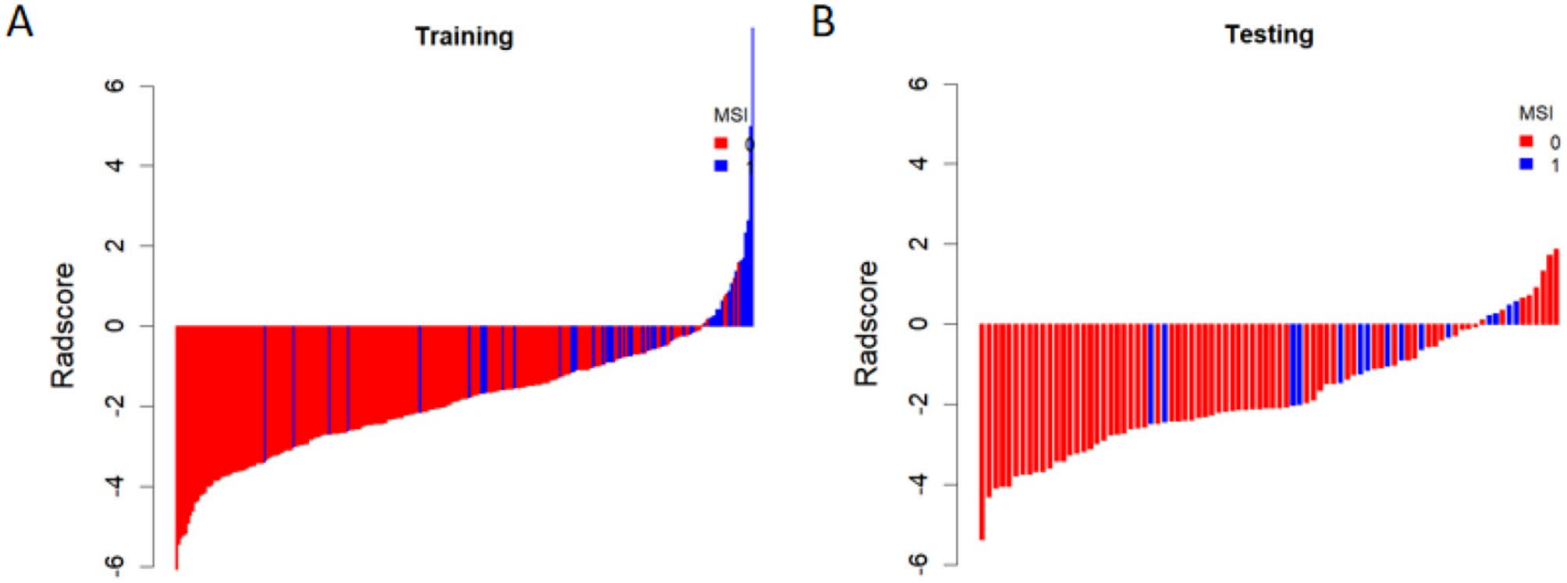
Waterfall plots of the arterial phase radscore for each patient in the training (**A**) and testing (**B**) datasets

**Fig. 3:**
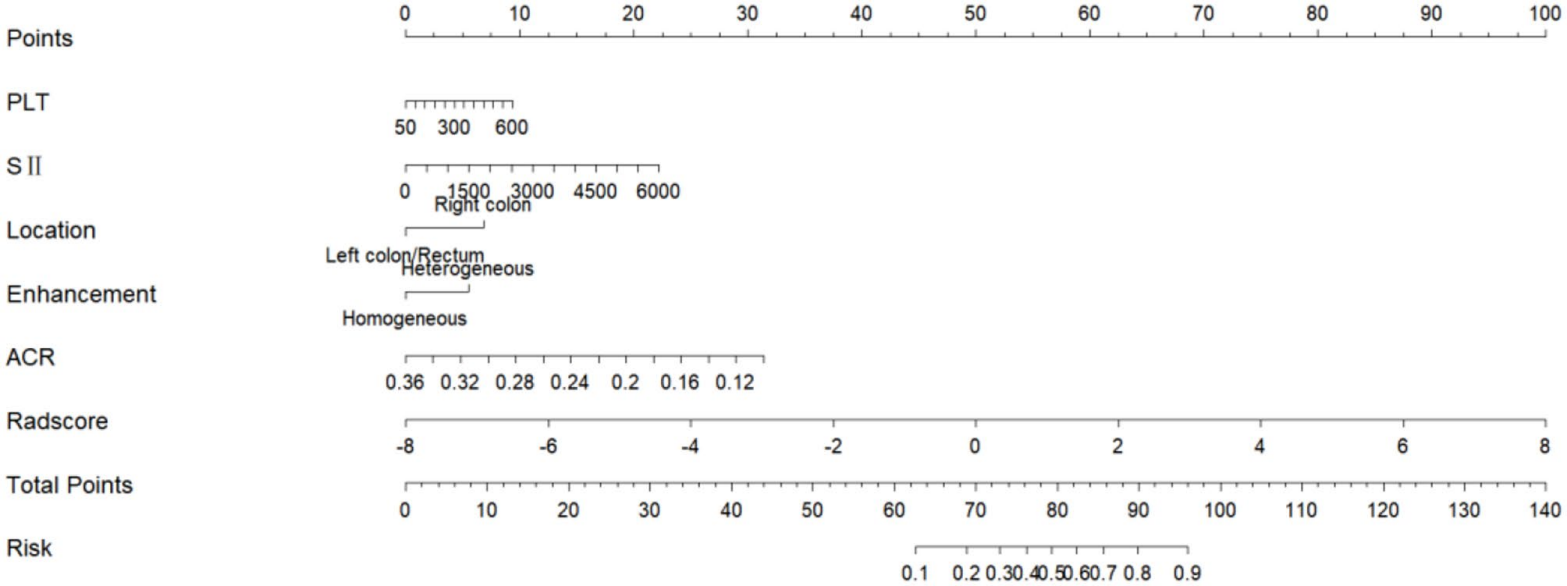
Nomogram for preoperative prediction of MSI status, consisting of five clinical-CT features and three-phase combination radscore

**Fig. 4:**
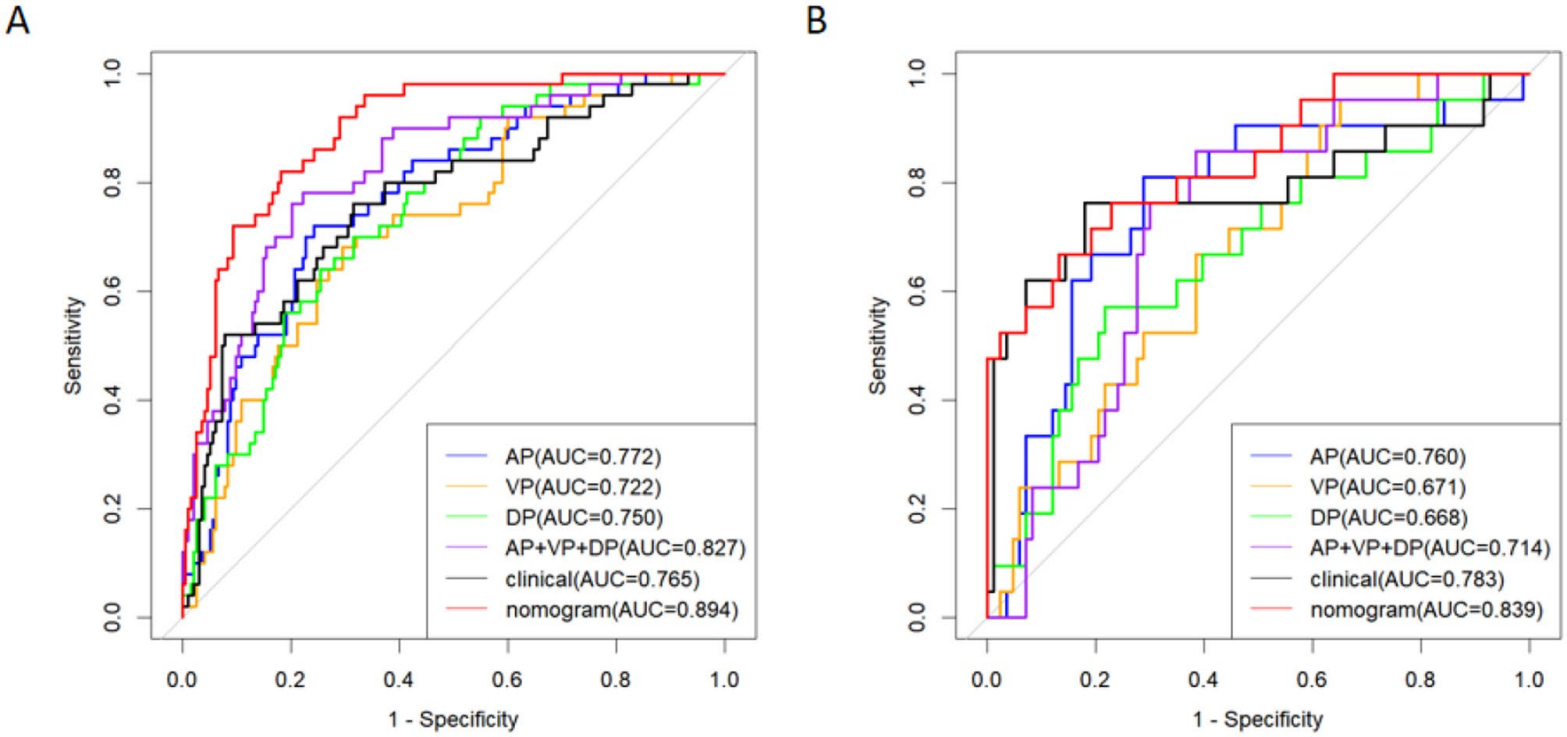
ROC curves of the prediction performance of six models in the training (**A**) and testing (**B**) datasets: AP model, VP model, DP model, three-phase combination model, clinical model, and nomogram model

The radscore is derived by combining the ten radiomics features screened by the optimal phase (three-phase combination) with the corresponding weights, as specified in the formula in Supplementary Material S1. This radscore was higher in the MSI group than in the MSS group in both the training and testing datasets (*P* < 0.05) (Fig. 2). The radscore and clinical CT features (PLT, SII, tumour location, enhancement pattern, and ACR) were combined to create a joint model and presented as a nomogram (Fig. 3). The results showed that the model had the best predictive efficacy, with an AUC of 0.894 (95% CI: 0.848–0.939) in the training dataset and 0.839 (95% CI: 0.738–0.940) in the testing dataset. The DeLong test showed that in the training dataset, there was a significant difference in the AUC of the nomogram model compared with the AUC of the AP, VP, DP and clinical models (*P* < 0.05), indicating that the nomogram model combining clinical-CT features and three-phase combination radiomics features could improve the predictive efficacy of MSI. The predictive efficacy of all models is detailed in Fig. 4; Table 4.

**Table 4:** Detailed performance of each model in the training and testing datasets

Datasets	Models	AUC(95% CI)	Sensitivity	Specificity	Accuracy	PPV	NPV
Training	AP model	0.772(0.701–0.843)	0.720	0.756	0.749	0.434	0.912
	VP model	0.722(0.645–0.799)	0.680	0.705	0.700	0.374	0.895
	DP model	0.750(0.679–0.821)	0.640	0.746	0.724	0.395	0.889
	AP + VP + DP model	0.827(0.763–0.891)	0.760	0.798	0.790	0.494	0.928
	Clinical model	0.765(0.687–0.843)	0.760	0.684	0.700	0.384	0.917
	Nomogram	0.894(0.848–0.939)	0.820	0.819	0.819	0.539	0.946
Testing	AP model	0.760(0.638–0.881)	0.810	0.711	0.731	0.415	0.937
	VP model	0.671(0.552–0.790)	0.952	0.349	0.471	0.270	0.967
	DP model	0.668(0.532–0.805)	0.571	0.783	0.740	0.400	0.878
	AP + VP + DP model	0.714(0.604–0.825)	0.857	0.614	0.663	0.360	0.944
	Clinical model	0.783(0.642–0.923)	0.762	0.819	0.808	0.516	0.932
	Nomogram	0.839(0.738–0.940)	0.868	0.867	0.827	0.560	0.911

AUC, Area under the curve; PPV, Positive predictive value; NPV, Negative predictive value

**Fig. 5:**
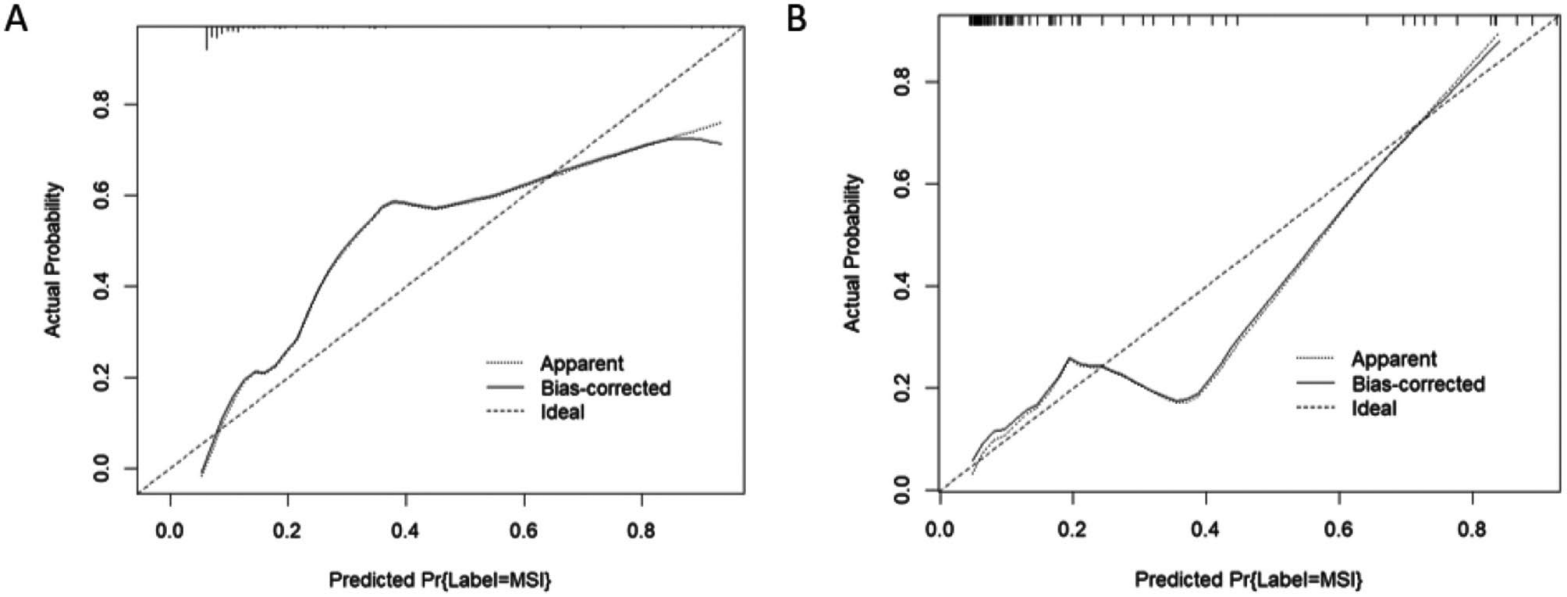
Calibration curves of the nomogram model in the training (**A**) and testing (**B**) datasets

Calibration curves (Fig. 5) indicate good agreement between the probability of predicting the MSI and the actual probability in the training and testing datasets. The Hosmer‒Lemeshow test for the nomogram model was not significant (*P* = 0.155 for the training dataset and *P* = 0.509 for the testing dataset), indicating that it did not deviate significantly from the ideal fit.

**Fig. 6:**
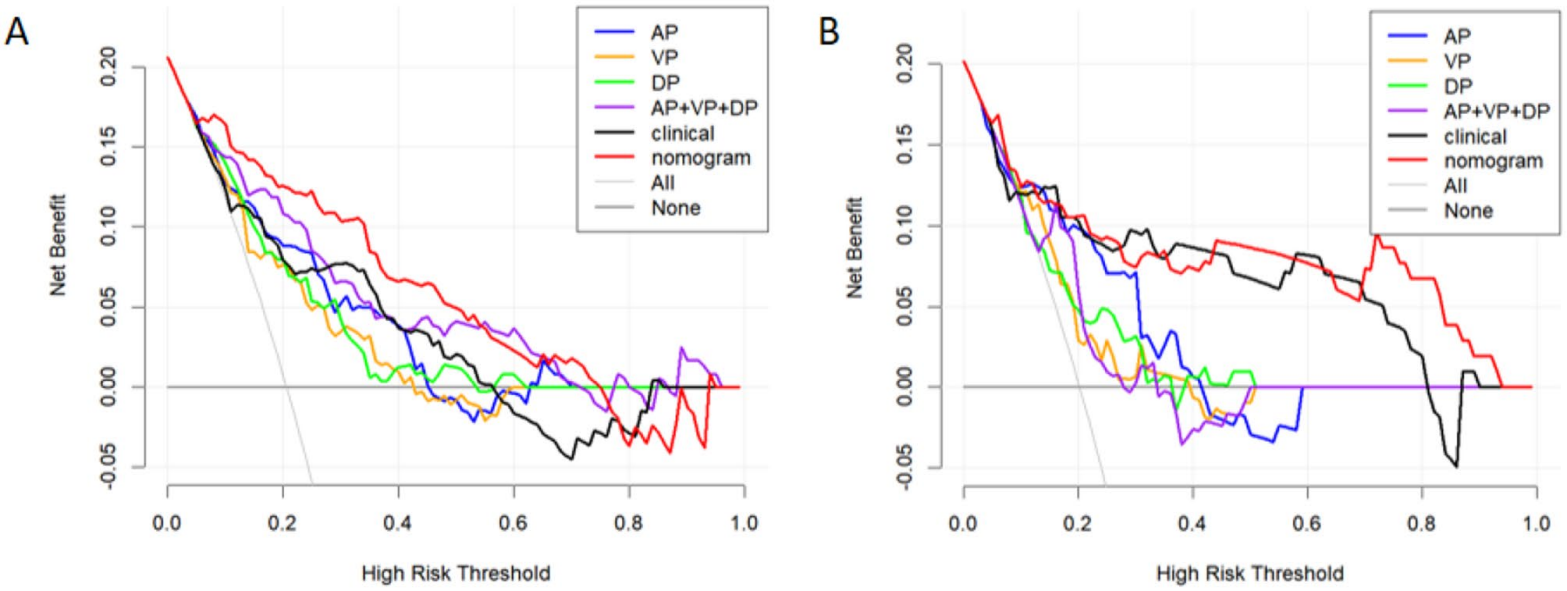
Decision curve analysis of each model in the training (**A**) and testing (**B**) datasets. The vertical coordinate indicates the net clinical benefit of the models, and the horizontal coordinate indicates the predicted threshold probability

DCA (Fig. 6) indicated that the nomogram model had a higher net benefit in differentiating MSI status in CRC patients within a reasonable range of threshold probabilities in both the training and testing datasets.

## Discussion

This study investigates the value of the nomogram model based on the combination of clinical-CT features and multiphasic enhanced CT radiomics for preoperative prediction of MSI status in CRC patients. The results show that the nomogram model combining clinical-CT features and three-phase combination radiomics features has better predictive efficacy in both the training and testing datasets.

The findings of the study demonstrated that among the included clinical features, PLT and SII were strongly correlated with the MSI status of CRC patients. In addition to its important function in haemostasis, PLT is an important inflammatory indicator [29], and SII is a comprehensive indicator based on NE, LYM, and PLT that has been proposed in recent years to better reflect the inflammatory response status of the body [30]. Chronic inflammation has been shown to be closely related to the key aspects of tumour development, recurrence, metastasis and immune escape [31, 32], and the inflammation and immune level of the body can be reflected by inflammation indices. Inflammation indices are usually obtained through blood routine and blood biochemistry tests, which are economical and relatively noninvasive tests for CRC patients [33]. The PLT level in the MSI group in this study was higher than that in the MSS group, which is consistent with the results of previous studies [17, 34]. Regarding the relationship between MSI and SII, which has not yet been reported, the present study found that the MSI group had a higher SII. This finding suggests that CRC patients with MSI status probably have more intense inflammatory responses. L De Smedt et al. [35] supported the view of this paper.

The findings of the study demonstrated that among the included CT features, tumour location, enhancement pattern, and ACR were strongly correlated with MSI status in CRC patients. Lesions with MSI status have a greater probability of occurring in the right colon, which is consistent with the findings of earlier research [36–38]. Currently, studies on the use of enhanced CT features for assessing MSI status in CRC patients have not been reported. Enhanced CT scanning is based on the formation of neovascularization of different tumours resulting in different haemodynamic changes to qualitatively analyse the lesions [39]. The CT value of the lesions is influenced by various factors, such as patient physiological factors, operator factors, and equipment factors [40]. To lessen the impact of these variables on the qualitative diagnosis, this study applies the CR for each period to standardize the CT values and more precisely evaluate the lesions. The results of this study revealed that the ACR of the MSI group was lower than that of the MSS group, and the lesion enhancement was more heterogeneous in MSI patients, which may be related to internal tumour necrosis and mucus components. The findings of Greenson JK et al. [41] support this view.

Previous studies have shown that CT radiomics has good predictive efficacy for MSI status in CRC patients. For example, Pei et al. [17] developed a combined clinical-VP CT radiomics nomogram model to predict the MSI status of CRC patients, and the results showed an AUC of 0.74 in the training group and 0.77 in the validation group. Jennifer S et al. [18] developed a combined clinical-VP CT radiomics model to predict MSI status in patients with stage II-III CRC. The results showed an AUC of 0.80 in the training group and 0.79 in the validation group. However, there are some limitations in these studies. First, only single-phase CT images were analysed, which could not reflect the features of dynamic changes in the tumour haemodynamics and could not comprehensively and holistically reflect the information of the tumour on the enhanced images. Second, none of these studies analysed enhanced CT features and did not combine comprehensive clinical features. Third, no corresponding treatment was taken for the imbalance in the incidence of MSI and MSS groups. In this study, based on triphasic enhanced CT images and comprehensive clinical-CT features, SMOTE was used to address the effect of imbalance in data distribution. The results indicated that the three-phase combination model outperformed the single-phase models, and the final nomogram model of the combined clinical-CT features and three-phase combination radiomics features had better predictive efficacy than the above mentioned studies, which can help with preoperative MSI status prediction in CRC patients.

The following limitations apply to our study. First, this study is a single-centre retrospective study. Although the nomogram model has good predictive efficacy, further validation in large-sample, prospective, and multicentre studies is needed. Second, manual segmentation is time-consuming, labour-intensive, and may be inaccurate due to the varying morphology of lesions. Third, the CT features analysed in this study, such as cT stage, cN stage, tumour length, maximum tumour diameter, enhancement pattern, and CR at each phase, are to some extent influenced by the radiologists’ experience and subjective factors. Fourth, this study hypothesized a lower ACR of lesions in the MSI group than in the MSS group, and the more heterogeneous enhancement of lesions in the MSI group might be related to components such as internal mucus and necrosis of the tumour. However, no further study of postoperative pathology was performed.

## Conclusion

This study demonstrates the good predictive efficacy of the nomogram model based on the combination of clinical-CT features and three-phase combination radiomics features, which is important for future clinical work. Thus, it can be used as an auxiliary tool for preoperative prediction of MSI status, for the development of treatment plans, and for the evaluation of prognosis in CRC patients.

### Electronic supplementary material

Below is the link to the electronic supplementary material.

Supplementary Material 1

Supplementary Material 2

Supplementary Material 3

## Data Availability

The datasets used and/or analysed during the current study are available from the corresponding author on reasonable request.
