# Supplementary material for: Preoperative prediction of microsatellite instability status in colorectal cancer based on a multiphasic enhanced CT radiomics nomogram model
Source: BMC Med Imaging. 2024 Apr 2;24:77. doi: 10.1186/s12880-024-01252-1 (PMC10988858; doi:10.1186/s12880-024-01252-1)
Supplement: Supplementary file 1 — Supplementary Material 1 [file 12880_2024_1252_MOESM1_ESM.docx]

Supplementary Material SⅠ. Formula for the calculation of Radscore.

Radscore=-1.30755+0.32765×A_exponential_glszm_SmallAreaEmphasis-0.56274×A_lbp-3D-m2_glcm_SumEntropy-0.40476×A_square_glszm_GrayLevelVariance+0.38555×A_wavelet-HLL_firstorder_90Percentile-0.50398×A_wavelet-HLH_firstorder_Mean-0.41084×A_wavelet-HHH_firstorder_Mean+0.50054×V_exponential_firstorder_Skewness+0.38556×V_lbp-2D_glszm_GrayLevelNonUniformityNormalized-0.38409×D_lbp-3D-m2_gldm_LargeDependenceLowGrayLevelEmphasis-0.57270×D_lbp-3D-k_gldm_SmallDependenceLowGrayLevelEmphasis+0.39385×D_squareroot_glszm_SmallAreaEmphasis+0.41460×D_wavelet-LLH_glszm_GrayLevelVariance
