# Supplementary figures and images for: Preoperative prediction of microsatellite instability status in colorectal cancer based on a multiphasic enhanced CT radiomics nomogram model
Source: BMC Med Imaging. 2024 Apr 2;24:77. doi: 10.1186/s12880-024-01252-1 (PMC10988858; doi:10.1186/s12880-024-01252-1)

Supplementary Material Figure SⅠ. Workflow of radiomics analysis.


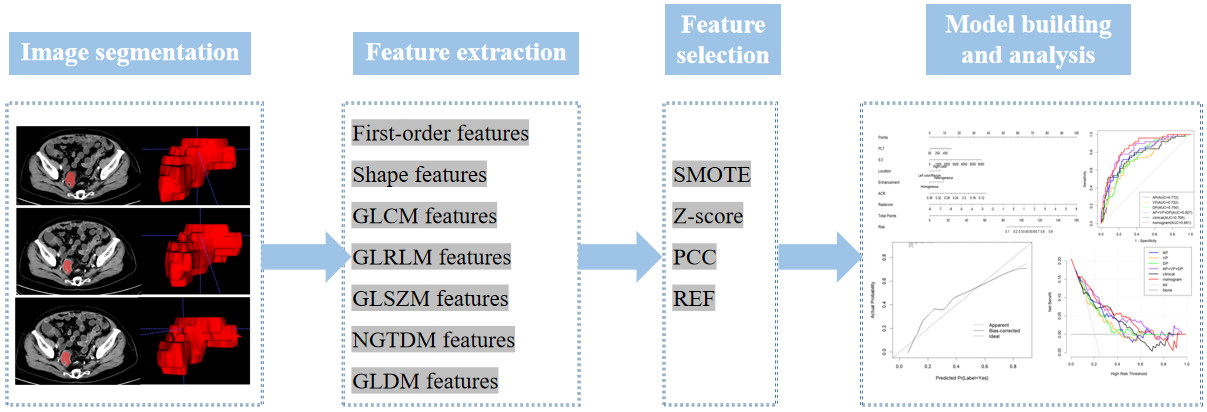

Supplement: Supplementary file 2 — Supplementary Material 2 [file 12880_2024_1252_MOESM2_ESM.docx]
