# Supplementary material for: Preoperative prediction of microsatellite instability status in colorectal cancer based on a multiphasic enhanced CT radiomics nomogram model
Source: BMC Med Imaging. 2024 Apr 2;24:77. doi: 10.1186/s12880-024-01252-1 (PMC10988858; doi:10.1186/s12880-024-01252-1)
Supplement: Supplementary file 3 — Supplementary Material 3 [file 12880_2024_1252_MOESM3_ESM.docx]

Supplementary Material Table SⅠ. The final radiomics features selected from each period.

| AP(n=10) | VP(n=7) | DP(n=7) | AP+VP+DP(n=12) |
| --- | --- | --- | --- |
| A_exponential_glszm_SmallAreaEmphasis | V_exponential_firstorder_Skewness | D_lbp-3D-m1_glszm_GrayLevelVariance | A_exponential_glszm_SmallAreaEmphasis |
| A_lbp-3D-m2_glcm_SumEntropy | V_lbp-2D_glszm_GrayLevelNonUniformityNormalized | D_lbp-3D-m1_glszm_HighGrayLevelZoneEmphasis | A_lbp-3D-m2_glcm_SumEntropy |
| A_original_glszm_SizeZoneNonUniformityNormalized | V_lbp-3D-m1_firstorder_Skewness | D_lbp-3D-m2_gldm_LargeDependenceLowGrayLevelEmphasis | A_square_glszm_GrayLevelVariance |
| A_square_firstorder_Variance | V_log-sigma-1-mm-3D_glcm_InverseVariance | D_lbp-3D-k_gldm_SmallDependenceLowGrayLevelEmphasis | A_wavelet-HLL_firstorder_90Percentile |
| A_square_glszm_GrayLevelVariance | V_wavelet-LLH_glcm_MCC | D_squareroot_glszm_SmallAreaEmphasis | A_wavelet-HLH_firstorder_Mean |
| A_squareroot_firstorder_Skewness | V_wavelet-HHH_firstorder_Median | D_wavelet-LLH_glszm_GrayLevelVariance | A_wavelet-HHH_firstorder_Mean |
| A_wavelet-HLL_firstorder_90Percentile | V_wavelet-LLL_glcm_MCC | D_wavelet-HHL_firstorder_Median | V_exponential_firstorder_Skewness |
| A_wavelet-HLH_firstorder_Mean |  |  | V_lbp-2D_glszm_GrayLevelNonUniformityNormalized |
| A_wavelet-HHL_firstorder_Mean |  |  | D_lbp-3D-m2_gldm_LargeDependenceLowGrayLevelEmphasis |
| A_wavelet-HHH_firstorder_Mean |  |  | D_lbp-3D-k_gldm_SmallDependenceLowGrayLevelEmphasis |
|  |  |  | D_squareroot_glszm_SmallAreaEmphasis |
|  |  |  | D_wavelet-LLH_glszm_GrayLevelVariance |
